# 853 Serial Casting for a Chronic Wound After an Electrical Burn: A Case Study

**DOI:** 10.1093/jbcr/iraf019.384

**Published:** 2025-04-01

**Authors:** Ingrid Malo-Leclerc, Zoë Edger-Lacoursière, Bernadette Nedelec, Stéphanie Jean

**Affiliations:** Villa Medica Rehabilitation Hospital and McGill University; Villa Medica Rehabilitation Hospital and McGill University; McGill University; University of Montreal

## Abstract

**Introduction:**

Serial casting (SC) is a non-invasive treatment for wound healing post burn. A recent review suggested that the mechanisms of action of plaster casts, pressure, occlusion and temperature rise, aid wound healing by influencing mechanotransduction and neurogenic inflammation. SC was applied to a 28-year-old male with a complex tibial wound from an electrical burn. Over 20 months, the patient underwent multiple unsuccessful treatments, including: 8 surgical debridements, 6 autographs and 2 local full thickness flaps, as well as various wound dressings, negative pressure wound therapy and low-level laser therapy. With no success, the patient and rehabilitation team decided to try SC.

**Methods:**

The purpose of the case study was to document the effect of SC on this patient’s chronic wound. A protective cast was made using 4 layers of synthetic cast padding beneath 8 layers of plaster of Paris, secured around the tibia by an elastic wrap. Wound dressing under the cast followed the physician’s instructions. The cast was changed every 3-7 days. The patient wore a custom fabricated orthosis to immobilize the ankle during the treatment period to reduce tension on the wound bed. Wound measurements were taken by the same therapist before casting and at each cast change, using a sterile cotton tip applicator. Additional observations on the wound, scar formation, and patient comfort were also recorded by the therapist.

**Results:**

Data collection is still ongoing as the wound has not yet closed. To date, the patient undergone SC for 3 months with a total of 15 casts. The initial wound was shaped as a non-symmetric ellipse with the following dimensions: 4 cm vertical, 0.8 cm horizontal proximal, 1 cm horizontal distal and 0.2 cm proximal depth and 0.3 cm distal depth. After the first cast, a wound size reduction of 5 & 50% was observed in the vertical and horizontal measurements. After one month, the dimensions had reduced by 20 & 63% and after 2 months, there was a 40 & 75% reduction. after several casts, the scar tissue surrounding the wound also became more supple and less adherent, resulting in reduced tension at the wound edges. Additionally, edema on the tibial zone substantially reduced. The casts were found both convenient and comfortable.

**Conclusions:**

Although data collection is ongoing, wound dimensions have reduced to an extend never achieved with previous treatments and substantial improvement of the surrounding scar scaring tissue has been observed.

**Applicability of Research to Practice:**

SC should be considered as a first-line conservative, non-invasive treatment for complex post-burn wounds. This case report has shown that this modality was effective for a patient who had tried various other unsuccessful options without any adverse effect.

**Funding for the Study:**

N/A

